# Innovative all‐in‐one exome sequencing strategy for diagnostic genetic testing in male infertility: Validation and 10‐month experience

**DOI:** 10.1111/andr.13742

**Published:** 2024-08-24

**Authors:** Manon S. Oud, Nicole de Leeuw, Dominique F. C. M. Smeets, Liliana Ramos, Godfried W. van der Heijden, Raoul G. J. Timmermans, Maartje van de Vorst, Tom Hofste, Marlies J. E. Kempers, Marijn F. Stokman, Kathleen W. M. D'Hauwers, Brigitte H. W. Faas, Dineke Westra

**Affiliations:** ^1^ Department of Human Genetics Radboud university medical center Nijmegen The Netherlands; ^2^ Department of Obstetrics and Gynaecology Radboud university medical center Nijmegen The Netherlands; ^3^ Department of Urology Radboud university medical center Nijmegen The Netherlands

**Keywords:** asthenozoospermia, azoospermia, diagnostics, gene panel, genetics, genetic testing, male infertility, oligozoospermia, teratozoospermia

## Abstract

**Background:**

Current guidelines indicate that patients with extreme oligozoospermia or azoospermia should be tested for chromosomal imbalances, azoospermia factor (AZF) deletions and/or *CFTR* variants. For other sperm abnormalities, no genetic diagnostics are recommended.

**Objectives:**

To determine whether exome sequencing (ES) with combined copy number variant (CNV) and single nucleotide variant (SNV) analysis is a reliable first‐tier method to replace current methods (validation study), and to evaluate the diagnostic yield after 10 months of implementation (evaluation study).

**Materials and Methods:**

In the validation study, ES was performed on DNA of patients already diagnosed with AZF deletions (*n* = 17), (non‐)mosaic sex chromosomal aneuploidies or structural chromosomal anomalies (*n* = 37), *CFTR* variants (*n* = 26), or variants in known infertility genes (*n* = 4), and 90 controls. The data were analyzed using our standard diagnostic pipeline, with a bioinformatic filter for 130 male infertility genes. In the evaluation study, results of 292 clinical exomes were included.

**Results:**

All previously reported variants in the validation cohort, including clinically relevant Y‐chromosomal microdeletions, were correctly identified and reliably detected. In the evaluation study, we identified one or more clinically relevant genetic anomalies in 67 of 292 of all cases (22.9%): these included aberrations that could have been detected with current methods in 30 of 67 patients (10.2% of total), (possible) (mono)genetic causes in the male infertility gene panel in 28 of 67 patients (9.6%), and carriership of cystic fibrosis in nine of 67 patients (3.1%).

**Conclusion:**

ES is a reliable first‐tier method to detect the most common genetic causes of male infertility and, as additional genetic causes can be detected, in our evaluation cohort the diagnostic yield almost doubled (10.2%–19.8%, excluding CF carriers). A genetic diagnosis provides answers on the cause of infertility and helps the professionals in the counseling for treatment, possible co‐morbidities and risk for offspring and/or family members. Karyotyping will still remain necessary for detecting balanced translocations or low‐grade chromosomal mosaicism.

## 1 INTRODUCTION

Genetic variation influences the reproductive potential of both males and females. The estimated contribution of currently tested genetic causes is 4%–9.2% of all cases of male infertility.^1^, ^2^, ^3^ Genetic causes are more common in severe forms of infertility such as non‐obstructive azoospermia (up to 25% explained by genetic factors) and specific subtypes such as multiple morphological abnormalities of the sperm flagellum (MMAF; up to 50% explained).^1^, ^4^

Routine diagnostic work‐up for genetic causes of male infertility recommended in the Dutch and the European guidelines includes karyotyping and azoospermia factor (AZF) deletion screening for patients with extreme oligozoospermia or azoospermia, and screening for variants in the *CFTR* gene in case of (assumed) congenital bilateral absence of the vas deferens (CBAVD).^5^, ^6^

The diagnostic yield of this combination of tests for azoospermia is approximately 20%–25%, while in extreme oligozoospermia (<1 million spermatozoa/mL), the diagnostic yield is approximately 10%.^1^, ^3^

Besides, these routinely tested genetic causes, pathogenic variants in at least 104 genes are confidently linked to male infertility, while at least 138 other genes are classified as candidate genes.^7^ Examining these genes for pathogenic variants could significantly improve the diagnostic yield of genetic testing, but has not yet found its way to routine diagnostics in the vast majority of laboratories due to cost‐efficiency concerns. Next‐generation sequencing methods are becoming more affordable and exome sequencing (ES), in which all protein‐coding regions of the genome are sequenced, is now routinely used in diagnostic genetic laboratories worldwide. In a recent study, ES in 1000 clinically diagnosed cases of non‐obstructive azoospermia identified a plausible monogenic cause in 20% of all patients.^8^ Furthermore, various recent studies in patient populations with extreme oligozoospermia or azoospermia without chromosomal aberrations, Y‐chromosome microdeletions or *CFTR* mutations, describe a monogenic diagnosis in 8.5%–15.7%.^9^, ^10^, ^11^, ^12^, ^13^

Next to the discovery of monogenic causes of infertility, ES data can also be used to detect copy number variations (CNVs). Deletions of the AZF regions were often difficult or impossible to detect using ES due to lack of exome enrichment targets in the protein‐coding genes located in these regions. However, new versions of exome enrichment kits do provide coverage of genes in the AZF regions, allowing the detection of imbalances in these regions. Further improvement of CNV detection methods also allows for reliable detection of aneuploidies of the autosomes and/or sex chromosomes. ES can therefore also be used to detect other common types of sex chromosomal anomalies in infertility such as Klinefelter syndrome (47,XXY) and structural aberrations of the Y‐chromosome (e.g., idic(Y)).

In this study, we describe the validation and implementation of an ES‐based strategy as a first‐tier method for genetic testing for male infertility. We propose a method to detect the classical deletions in the AZFa, AZFb, and/or AZFc region using ES and determine the sensitivity and specificity of our test to detect sex chromosomal anomalies, AZF deletions and *CFTR* variants. Furthermore, we demonstrate the diagnostic yield of our strategy in the first 10 months after implementation in our laboratory.

## 2 MATERIALS AND METHODS

### 2.1 Patients, samples, and statistics

For the validation study, a total of 57 patients with a genetic diagnosis through routine diagnostic methods including karyotyping, AZF deletion screening with sequence‐tagged sites primers (STS‐PCR), *CFTR* screening for the most common European mutations (CF‐EU2v1 kit, Elucigene Diagnostics) and/or *CFTR* Sanger sequencing or multiplex ligation probe amplification (MLPA) (SALSA MLPA Probemix P091 CFTR, MRC Holland) were included as positive control for aberrations essential to be detected (“mandatory” aberrations). Furthermore, 27 additional positive controls, with aberrations optional for detection, but not included in current guidelines (“optional” aberrations: mosaic (sex) chromosomal abnormalities, partial AZF deletions, *CFTR* variants not present in the CF‐EU2v1 kit, single nucleotide variant [SNVs], or CNVs in known infertility genes), and 90 negative controls (with a normal male karyotype, negative STS‐PCR, and/or negative CF‐EU2v1 kit in routine diagnostics) were incorporated. All positive control samples could be used as a negative control in a technique that revealed a normal diagnostic result (e.g., a 47,XXY karyotype with a normal STS‐PCR for the AZF regions, is a negative control for the AZF deletions). An overview of the positive and negative controls used in the validation study is given in Tables SI and SII.

For the evaluation study, all patients (*n* = 292) that received ES for male infertility in the Division of Genome Diagnostics of the Department of Human Genetics of the Radboud University Medical Center from March 2023 until December 2023 were included (Table SIII). The test was available to all patients with (non‐)obstructive azoospermia, oligozoospermia (<5 million spermatozoa per mL semen) or extreme oligozoospermia (<1 million spermatozoa per mL semen), globozoospermia, macrozoospermia, MMAF, other types of extreme asthenozoospermia or teratozoospermia (according to WHO, 2021^14^), and cases with fertilization failure after intracytoplasmic sperm injection (ICSI). The average age (at time of request) was 34.7 years (24.3–61.2 years); ethnicity and geographic origin was not available, as this specific background information is not needed to perform genetic diagnostics in our laboratory. A total of 252 of 292 (86.3%) received karyotype analysis next to ES.

We used the “rule of three” to calculate sensitivity and sensitivity, which states, at a 95% confidence interval, that the probability of a certain event that is not seen in validation of sample size *n* is 3/*n*.^15^

### 2.2 Exome enrichment, sequencing, and variant calling

DNA was isolated from EDTA blood by standard procedures. For the validation study, exomes were enriched using the Twist Human Exome + RefSeq Panel Kit (Twist Bioscience). For the evaluation study, the updated Twist Exome 2.0 plus Comprehensive Exome Spike‐in Kit (Twist Bioscience) was used. For all samples, 2 × 150‐basepair (bp) paired‐end sequencing using the Illumina NovaSeq 6000 (Illumina) short‐read sequencing platform was performed, aiming at an average coverage of 100×. Downstream processing of the ES data and variant calling was performed using an in‐house pipeline as described previously.^16^ Read alignment was done with Burrows‐Wheeler Aligner (BWA2 v2.2.1). For SNV calling, GATK HaplotypeCaller (v3.8) was used. CNV calling was performed using two algorithms specifically developed for exome data. CoNIFER (v0.2.0), an algorithm accurately detecting CNVs affecting three exons or more, was used to detect larger deletions and gains on the autosomes and chromosome X.^17^ ExomeDepth (v1.1.10), which has a lower specificity than CoNIFER, but a higher resolution because of CNV detection in 170 bp windows, was used to detect single exon deletions, (partial) aneuploidies, and deletions on the Y chromosome.^18^ CNV profiles were visualized in the Integrative Genomics Viewer (IGV; v2.15.4).^19^ Subsequentially, variants outside the genes of the male infertility gene panel (see below and in Table SIV) were (bioinformatically) filtered out and remaining variants were interpreted according to the practice guideline of the Dutch Society for Clinical Genetic Laboratory Diagnostics (VKGL) and the Association for Clinical Genetic Science (ACGS).^20^

### 2.3 Exome analysis workflow for male infertility

**FIGURE 1:**
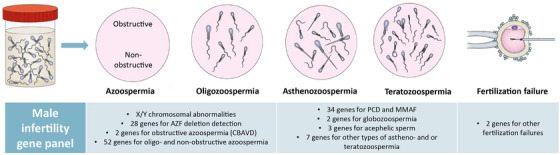
A total of 130 genes were included in the first diagnostic panel version (DG‐3.6), with genes associated with azoospermia, oligozoospermia, asthenozoospermia, teratozoospermia, and fertilization failure. An overview of all genes and their associated phenotype is given in Table SIV. AZF, azoospermia factor; CBAVD, congenital bilateral absence of the vas deferens; MMAF, multiple morphological abnormalities of the sperm flagella; PCD, primary ciliary dyskinesia. Figure adapted from.^42^

We built an analysis workflow to detect the various types of genetic variation underlying male infertility (Supporting Information File). Data analysis included CNV analysis to detect numerical chromosomal anomalies or aneuploidies (with CoNIFER and ExomeDepth), CNV calling on genes located in AZF regions, as well as interpretation of SNVs and CNVs within our virtual male infertility gene panel consisting of 130 genes (Figure 1 and Table SIV).

**FIGURE 2:**
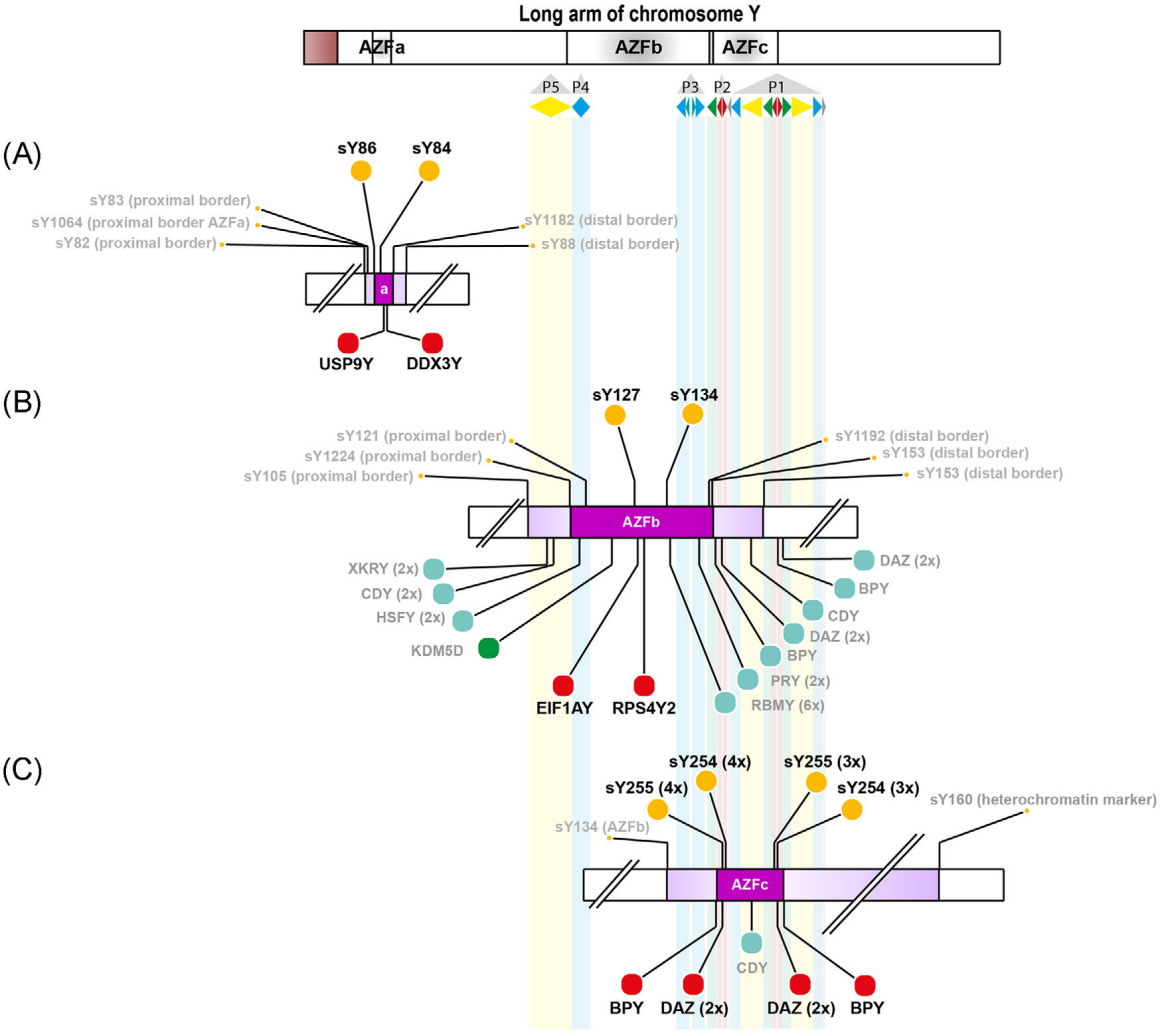
Schematic overview of the azoospermia factor (AZF) regions, sequence‐tagged sites (STS sites), and genes on the Y chromosome. The Y chromosome, and especially the AZFb and AZFc region, contains highly repetitive sequences. A graphic representation of the sequence organization is presented below the ideogram depicting the Y chromosome. Five palindromes (P1–P5) containing various amplicon sequence families (yellow, blue, turquoise, green, red, and gray) are depicted.^28^ For reference, transparent bands outline the amplicons in the background. The orange dots represent the STS sites used for detection of the classic AZFa (A), AZFb (P5/proximal P1; B) and AZFc (b2/b4; C) deletions according to the EAA/EMQN best practice guidelines (2014 and 2023).^24^, ^26^ In dark purple, the critical deletion region, and in lighter purple, the region in which the breakpoints are typically located. The large circles above the regions indicate the primary STS markers (two per region) used for deletion detection and the small circles indicate the STS markers used for the extension analysis. Below in red, the suggested genes for detection of deletion of the respective region. The other genes which can be used for extension analysis are depicted in green (single‐copy gene) and teal (multicopy genes). For an overview in tabular form, see Table SV.

To replace STS‐PCR testing, we explored ways to detect AZF deletions in ES data. The AZF regions contain multiple protein‐coding and non‐coding RNA genes, of which all protein‐coding genes are covered in the Twist Bioscience “Human Comprehensive Exome” and “X2 comprehensive exome” kit (Twist Bioscience). Analysis of the AZF STS and protein‐coding gene sites reveals that each of the AZFa, ‐b, or ‐c deletions results in the complete loss of all copies of at least two genes (Figure 2 and Table SV). As such, detection of a complete loss of *USP9Y* and *DDX3Y* in the AZFa region, *EIF1AY* and *RPS4Y2* in the AZFb region, and *BPY* and *DAZ* in the AZFc region can be used to detect classical AZF deletions and is expected to give similar precision to the STS site deletions. Partial AZF deletions such as gr/gr and b2/b3 do not cause complete loss of the same copies and can therefore be discriminated from the “classical” complete AZF deletions.

### 2.4 Gene panel

The gene panel is based on an earlier published systematic review of the clinical validity of gene–disease relationships^7^; genes with a score of at least 7 out of 17 points were included. A literature search was performed to include more recently published genes (until March 2023). The used gene panel (version DG‐3.6, https://order.radboudumc.nl/genetics) consists of 130 genes (Figure 1 and Table SIV), of which 52 are primarily associated with an oligo‐ or (non‐obstructive) azoospermia phenotype, two genes with CBAVD, 34 genes with MMAF/primary ciliary dyskinesia (PCD), three genes with acephalic spermatozoa, two genes with globozoospermia, two genes with fertilization failure (male factor), and seven other genes linked to astheno‐ and/or teratozoospermia. A total of 28 genes were included for accurate detection of AZF deletions because of their localization in the AZFa, AZFb, or AZFc region. Analysis of the complete *CFTR* gene was performed in all patients, even without suspicion of CBAVD, as it is known that CBAVD can be missed on physical examination.^21^ Carriership of (classical) cystic fibrosis (CF) was reported in all cases, because of the possible consequences for the chosen medically assisted reproduction (MAR) trajectory for a carrier couple.

### 2.5 Ethical background

The methods of this study were approved by the institutional review board of the Radboud University Medical Center (number 2011‐188 and 2020‐7142) and the study is in compliance with relevant guidelines and regulations of the Declaration of Helsinki. De‐identified data from clinical samples can be used by a diagnostic genetic laboratory to evaluate novel diagnostic procedures and derived clinically relevant variants can be shared.

## 3 RESULTS

### 3.1 Validation of the ES strategy for “mandatory” variants

In order to be able to use the ES strategy as a first‐tier method for male infertility, the sensitivity and specificity of the analysis strategy should exceed 95% for each type of the following “mandatory” anomalies mentioned in current (inter)national guidelines for genetic testing in male infertility^5^, ^6^: classical AZF deletions, non‐mosaic sex chromosomal anomalies, and the most common European *CFTR* variants present in the Elucigene CF‐EU2v1 kit.

For the detection of classical complete AZF deletions, 24 positive and 37 negative controls were tested: AZFa (*n* = 2), AZFb (P5/proximal P1; *n* = 2), AZFbc (P5/distal P1; *n* = 2), AZFc (b2/b4; *n* = 11), and AZF deletions due to chromosomal anomaly (i.e., idic(Y), *n* = 7; Table SI). The results of ES were 100% concordant with the results of the STS‐PCR previously performed. With the “rule of three^15^” the sensitivity and specificity of the ES strategy was at least 95.1% for detection of the classical complete AZF deletions (Table SII).

For the detection of non‐mosaic sex chromosomal anomalies, 22 positive and 51 negative controls were tested: 47,XXY (*n* = 10), 47,XYY (*n* = 5), 46,XX (SRY+) (*n* = 3), 46,X,idic(Y)(q11) (*n* = 2), and 46,X,iso(Y) (*n* = 2). The results of ES were 100% concordant with the results of the karyotyping, (fluoresence in situ hybridization (FISH) and/or array analysis previously performed, although in four cases with an idic(Y) or iso(Y), there were differences in the established breakpoint. Using the “rule of three,” the sensitivity and specificity of the ES strategy was at least 95.9% for detection of non‐mosaic sex chromosomal anomalies.

For the detection of common *CFTR* variants present in the Elucigene CF‐EU2v1 kit, a total of 18 positive and 83 negative controls were used. In 17 of 18 positive controls, the results of the exome analysis matched the results that were obtained earlier (sensitivity 94%), including the length of the T/TG‐stretch in intron 9 of the gene (NM_00492.4).^22^ None of the negative controls harbored a variant present in the Elucigene CF‐EU2v1 kit based on ES (specificity 100%). The variant that was missed by ES was located in intron 22 (NM_000492.4:c.3718‐2477C>T) and not covered by the exome targets in the Twist Human Exome + RefSeq Panel Kit. The Elucigene kit also contains another deep‐intronic variant (NM_000492.4:c.1680‐886A>G) not covered by the same exome enrichment kit, but this variant was not tested in this validation study. The updated version of the exome enrichment kit (Twist Exome 2.0 plus Comprehensive Exome Spike‐in Kit; Twist‐X2) does provide coverage for both regions, with an average coverage well above the minimally required 20× for accurate variant detection (internal validation; unpublished data). As our laboratory switched to the Twist‐X2 enrichment kit before implementation of the ES strategy, using the “rule of three” we determined that the sensitivity and specificity of the ES strategy for *CFTR* variants was at least 97%.

### 3.2 Validation of the ES strategy for “optional” variants

In addition to replacing the tests for the above mentioned “mandatory” variants, we explored the possibilities of the exome analysis strategy for recognizing “optional” variants, such as mosaic sex chromosomal anomalies, partial AZF deletions, *CFTR* variants outside the Elucigene kit, and CNVs of (parts of) male infertility genes. These variants regularly occur in male infertility, but are not included in current guidelines. As our ES strategy was not designed for the detection of these variants, no formal sensitivity or specificity was calculated for these variants.

We tested a total of 11 mosaic sex chromosomal anomalies including 45,X/46,XY (*n* = 3), 47,XXY/46,XY (*n* = 2), and various other mosaic anomalies (*n* = 6), with a level of mosaicism ranging from 14% to 87% (abnormal cells; Table SI). In all cases, an abnormal CNV profile was seen, matching the original diagnostic outcome. However, in two cases, the anomaly appeared to be present in all cells rather than mosaic. Furthermore, although ES revealed an abnormal CNV profile, the analysis was unable to accurately detect a mos 45,X[17]/46,X,idic(Yq11.221)[13], as it appeared to be a terminal AZFbc deletion: with this mosaic grade, the short arm and part of the long arm of the Y‐chromosome are present in about 50% of the DNA, resulting in a seemingly normal male profile for Yp11.32q11.221. In the negative control samples (all 46,XY after karyotyping), we unexpectedly identified one case of 45,X/46,XY based on the exome analysis (VAL84). This sample apparently lost the Y‐chromosome in approximately 30% of the DNA, but this mosaicism was not detected by conventional karyotyping (performed in 2012), in which only five metaphases were analyzed.

In addition to the classical, complete, AZF deletions, a wide range of rearrangements of the AZF region has been described, including duplication of the AZF regions or partial AZF deletions in which several, but not all copies of multicopy genes located in the AZFb and AZFc region are deleted.^23^ Partial deletions, such as gr/gr, are risk factors for male infertility, but the clinical significance of these variants remains uncertain.^24^ To investigate the detection performance for other types of AZF rearrangements using ES, we included four samples with such rearrangements (gr/gr, b2/b3, and b1/3 deletions and AZFbc duplication). The AZFbc duplication was detected by the analysis software, while the three different types of deletions were not detected by the analysis software nor was visual inspection sufficiently reliable for detection.

Lastly, we also included a total of 12 samples with anomalies currently not detected by routine diagnostic techniques: five samples with a *CFTR* SNV not included in the Elucigene kit, three samples with an intragenic *CFTR* deletion, three samples with a SNV in a known infertility gene (*M1AP*, *TEX14*, and *DNAH1*) and one sample with a homozygous *DPY19L2* deletion. In all cases, the variant was correctly detected.

### 3.3 Results in first 292 diagnostic patients

After the validation study, we implemented the male infertility ES strategy in our routine diagnostic laboratory. All patients in the evaluation cohort (*n* = 292) received the complete analysis protocol including AZF deletion screening, screening for sex chromosomal anomalies, and SNV/CNV detection in the genes in the male infertility gene panel. Simultaneously, karyotyping analysis was recommended to rule out balanced translocations and mosaicisms (>20% abnormal cells). A total of 252 of 292 (86.3%) received karyotype analysis next to ES (Table SIII).

**FIGURE 3:**
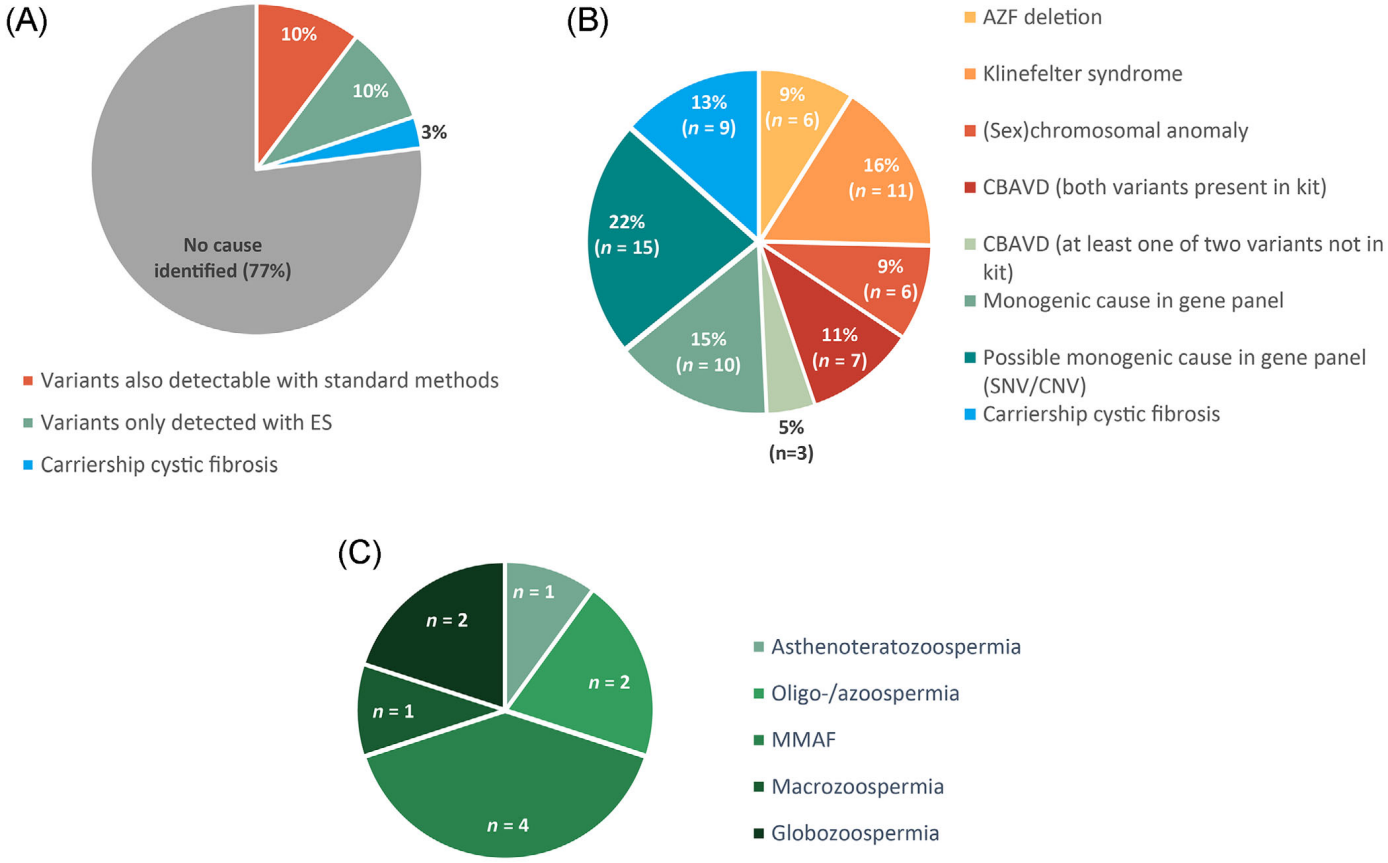
Diagnostic results of exome sequencing (ES) in the implementation cohort. In A, the overall results of 292 infertile males are shown, with the clinically relevant variants divided into variants that could have been detected with karyotyping, AZF deletion screening, or *CFTR* analysis (CF‐EU2v1 kit, Elucigene Diagnostics), variants that are not detectable with those techniques, and carriership for cystic fibrosis (CF). These clinically relevant variants are further elucidated in B, while the phenotypes associated with the genes in which a monogenetic cause was identified with ES, other than CBAVD, are shown in C. AZF, azoospermia factor; CBAVD, congenital bilateral absence of the vas deferens; CNV, copy number variant; MMAF, multiple morphological abnormalities of the sperm flagella; SNV, single nucleotide variant.

In 67 of 292 of all cases (22.9%), we identified one or more (potentially) clinically relevant genetic anomalies (Figure 3). As shown in Table 1A, 17 of these (5.8% of total; 25.4% of abnormal) harbored an abnormal CNV profile indicating a (sex) chromosomal anomaly (47,XXY: *n* = 11; idic(Y) in mosaic: *n* = 2, 46,XX‐male: *n* = 3, complex translocation: *n* = 1), which were all detected with karyotyping as well; no sex chromosomal anomalies were missed with ES. Six cases (2.1% of total; 8.9% of abnormal) had a complete AZF deletion: one with a complete AZFb (P5/proximal P1) deletion and five with a complete AZFc (b2/b4) deletion.

In 10 patients (3.4% of total; 14.9% of abnormal), two variants in *CFTR* were identified that were associated with CBAVD and/or mild classical CF; in three of 10, at least one rare variant was detected that is not present in the Elucigene CF‐EU2v1 kit and would not have been identified without sequencing the complete gene. Remarkably, in only three of nine (33.3%) patients for which results of physical examination and ejaculate volume and/or pH were available, the vas deferens were not palpable at physical examination; in the remaining the vas deferens were said to be present or doubtfully palpable; in nine of nine (100%) the semen analysis showed azoospermia, low volume (<1.5 mL), and low pH (<7.2; Table 2), in line with the CBAVD expected pattern.

**TABLE 1A:** Positive results of the implementation study, with variants also detectable with the standard methods.

Patient	Phenotype (as provided at request)	Result exome sequencing	Conclusion	Karyotyping result
**AZF deletions**				
P39	Azoospermia	seq[GRCh37] Yq11.222q11.223(20708557_24564028)×0 NC_000024.9:g.(15032390_20708557)_(24564028_26979967)del	AZFb deletion (P5/proximal P1)	Not performed
P51	Infertility	seq[GRCh37] Yq11.223q11.23(25130410 _27198251)×0 NC_000024.9:g.(24564028_25130410)_(27198251_58911807)del	AZFc deletion (b2/b4)	46,XY
P53	Azoospermia	seq[GRCh37] Yq11.223q11.23(25130410 _27198251)×0 NC_000024.9:g.(24564028_25130410)_(27198251_58911807)del	AZFc deletion (b2/b4)	46,XY
P122	Azoospermia	seq[GRCh37] Yq11.223q11.23(25130410 _27198251)×0 NC_000024.9:g.(24564028_25130410)_(27198251_58911807)del	AZFc deletion (b2/b4)	46,XY
P135	Infertility	seq[GRCh37] Yq11.223q11.23(25130410 _27198251)×0 NC_000024.9:g.(24564028_25130410)_(27198251_58911807)del	AZFc deletion (b2/b4)	Not performed
P259	Azoospermia	seq[GRCh37] Yq11.223q11.23(25130410 _27198251)×0 NC_000024.9:g.(24564028_25130410)_(27198251_58822656)del	AZFc deletion (b2/b4)	46,XY
**Klinefelter syndrome**				
P54	Azoospermia	seq(X)×2,(Y)×1 NC_000023.10:g.[pter_qter]sup	Klinefelter syndrome	47,XXY
P64	Azoospermia	seq(X)×2,(Y)×1 NC_000023.10:g.[pter_qter]sup	Klinefelter syndrome	47,XXY[28]/46,XY[2]
P67	Azoospermia	seq(X)×2,(Y)×1 NC_000023.10:g.[pter_qter]sup	Klinefelter syndrome	Not performed
P100	Infertility	seq(X)×2,(Y)×1 NC_000023.10:g.[pter_qter]sup	Klinefelter syndrome	Not performed
P185	Infertility	seq(X)×2,(Y)×1 NC_000023.10:g.[pter_qter]sup	Klinefelter syndrome	47,XXY
P205	Azoospermia	seq(X)×2,(Y)×1 NC_000023.10:g.[pter_qter]sup	Klinefelter syndrome	47,XXY
P206	Infertility	seq(X)×2,(Y)×1 NC_000023.10:g.[pter_qter]sup	Klinefelter syndrome	47,XXY
P226	Azoospermia	seq(X)×2,(Y)×1 NC_000023.10:g.[pter_qter]sup	Klinefelter syndrome	47,XXY
P279	Infertility	seq(X)×2,(Y)×1 NC_000023.10:g.[pter_qter]sup	Klinefelter syndrome	47,XXY
P284	Azoospermia	seq(X)×2,(Y)×1 NC_000023.10:g.[pter_qter]sup	Klinefelter syndrome	47,XXY
P291	Azoospermia	seq(X)×2,(Y)×1 NC_000023.10:g.[pter_qter]sup	Klinefelter syndrome	47,XXY
**(Sex) chromosomal anomalies**				
P55	Azoospermia	seq[GRCh37] Ypterq11.221(2655082_15032390)×1∼2,Yq11.221qter(19880860_qter)×0 NC_000024.9:g.pter_(15032390_19880860)dup mos NC_000024.9:g.(15032390_19880860)_qterdel	idic(Y) in mosaic	mos 46,X,idic(Y)(q11.22)[19]/45,X[13]
P214	Infertility	seq[GRCh37] Xp22.33q28(3544582×1,3559783_qter×2), Yp11.2q12(5605902×1,6114298_qter×0) NC_000023.10:g.(3544582_ 3559877)_qterdup NC_000024.9:g.(5605902_6114298)_qterdel	XX‐male (SRY+), most probably due to t(X;Y)(p22.33;p11.2)	46,XX.ish der(X)t(X;Y)(p22.33;p11.2)(SRY+)
P241	Azoospermia	seq[GRCh37] 4p11q12(48906608×2,48988393_52887490×1, 52887882×2),4q12(54374394×2,54424011_55141218×1,55143475×2),4q12(55606853×2,55946107_57220377×1,57220849×2) NC_000004.11:g.[(48906608_48988393)_(52887490_52887882)del;(54374394_54424011)_(55141218_55143475)del; (55606853_55946107)_(57220377_57220849)del]	complex CNV profile	45,XY,der(4)t(4;21)(p10;q10), der(22)t(4;22)(q10;q10)
P254	Infertility	seq[GRCh37] Xp22.33q28(3264653×1,3530228_qter×2), Yp11.2q12(5605902×1,6114298_qter×0) NC_000023.10:g.(3264653_3530228)_qterdup NC_000024.9:g.(5605902_6114298)_qterdel	XX‐male (SRY+), most probably due to t(X;Y)(p22.33;p11.2)	46,XX.ish der(X)t(X;Y)(p22.33;p11.2)(SRY+)
P269	Azoospermia	seq[GRCh37] Ypterq11.222(pter_19991724)×2,Yq11.222qter (20138043_qter)×0 NC_000024.9:g.pter_(19991724_20138043)dup NC_000024.9:g.(19991724_20138043)_qterdel	idic(Y) in mosaic	mos 46,X,idic(Y)(q11.222)[32]/45,X[3]
P292	Azoospermia	seq[GRCh37] Xp22.33q28(2699968_qter×2),Yp11.2q12(2848037×1, 3447310_qter×0) NC_000023.10:g.(pter_2699968)_qterdup NC_000024.9:g.(2848037_3447310)_qterdel	XX‐male (SRY+), most probably due to t(X;Y)(p22.33;p11.3)	46,X,der(X)t(X;Y)(p22.33;p11.3).ish der(X)t(X;Y)(SRY+)

For CFTR‐related CBAVD patients, see Table 2.

Abbreviations: AZF, azoospermia factor; CNV, copy number variant.

**TABLE 1B:** Positive results of the implementation study with (likely) pathogenic variants identified by exome sequencing in male infertility genes, giving a monogenic cause of the fertility problem.

Patient	Phenotype (as provided at request)	Result exome sequencing	Associated phenotype (inheritance) (OMIM phenotype)
P1	Globozoospermia	seq[GRCh37] 12q14.2q14.2(63954291_64062599)×1 (het. deletion *DPY19L2*) NC_000012.11:g.(63544616_63954291)_(64062599_64173740)del *DPY19L2* Chr12(GRCh37):g.64011119del NM_173812.5:c.1183del p.(Ser395fs); hem.	globozoospermia (AR) (OMIM #613958; spermatogenic failure 9)
P15	Infertility	*CFAP69* Chr7(GRCh37):g.89934091_89934092dup NM_001039706.3:c.2222_2223dup p.(Phe742fs); hom.	MMAF (AR) (OMIM #617959; spermatogenic failure 24)
P61	Infertility	*NR5A1* Chr9(GRCh37):g.127262607T>C NM_004959.5:c.632A>G p.(Tyr211Cys); het. pat.	oligo/azoospermia (AD) (OMIM #613957; spermatogenic failure 8; variable expression and incomplete penetrance described)
P110	Macrozoospermia	*AURKC* Chr19(GRCh37):g.57743441del NM_001015878.2:c.145del p.(Leu49fs); hom.	macrozoospermia (AR) (OMIM #243060; spermatogenic failure 5)
P130	Oligoasthenozoospermia	*FSIP2* Chr2(GRCh37):g.186659725del NM_173651.4:c.7862del p.(Leu262fs); hom.	MMAF (AR) (OMIM #618153; spermatogenic failure 34)
P179	Asthenozoospermia	*DNAI1* Chr9(GRCh37):g.34459053dup NM_012144.4:c.48+2dup r.spl p.?; het. *DNAI1* Chr9(GRCh37):g.34512333A>G NM_012144.4:c.1402‐2A>G r.spl p.?; het.	MMAF (AR) (OMIM #244400; ciliary dyskinesia, primary 1, with or without situs inversus)
P217	Azoospermia	*RBBP7* ChrX(GRCh37):g.16871956_16871959del NM_002893:c.607_610del p.(Leu203fs); hem.	oligo/azoospermia (XLR) (PMID 35809576)
P222	Globozoospermia	seq[GRCh37] 12q14.2q14.2(63954291_64062173)×0 (hom. deletion *DPY19L2*) NC_000012.11:g.(63544616_63954291)_(64062173_64174722)del; hom.	globozoospermia (AR) (OMIM #613958; spermatogenic failure 9)
P256	Azoospermia	seq[GRCh37] 15q15.3(43875869×2,43888165_43910888×1,43922745_43941195×0, 43987997_43991398×1,44038651× 2) (comp. het. deletions *CATSPER2*) NC_000015.9:g.[(43875869_43888165)_(43991398_44038651)del];[(43910888_43922745)_(43941195_43987997)del]	asthenozoospermia (AR) (OMIM #611102; deafness and male infertility)
P274	Infertility	*CFAP43* Chr10(GRCh37):g.105928535C>T NM_025145.7:c.2658G>A p.(Trp886*); hom.	MMAF (AR) (OMIM #617592; spermatogenic failure 19)

For *CFTR*‐related CBAVD patients, see Table 2; for possible monogenic cause in the gene panel or beyond, see Table SIII.

Abbreviations: AD, autosomal dominant; AR, autosomal recessive; comp.het., compound heterozygous; hem, hemizygous; het., heterozygous; hom, homozygous; MMAF, multiple morphological abnormalities of the sperm flagella; OMIM, Online Mendelian Inheritance in Man; pat., paternal.

In nine patients (3.1% of total; 13.4% of abnormal) only one (likely) pathogenic variant associated with CF was detected in *CFTR*, indicating carriership of CF; the subjects with azoospermia had normal volume and/or pH, arguing against a possible missed CBAVD. This carriership frequency of one of 31 (nine of 282 non‐*CFTR*‐related CBAVD patients) is comparable with the carriership frequency in the general Dutch population (incidence CF one of 3600, carriership frequency one of 30). In all patients with one or more (likely) pathogenic *CFTR* variant, carriership analysis in the partner was offered: in 14 of 19 this was performed, and all these (Dutch) partners had a negative Elucigene CF‐EU2v1 kit, reducing the risk for the couple of having an affected child to one of 1200 (for men with two (likely) pathogenic variants) and one of 2400 (for men with one (likely) pathogenic variant) in each pregnancy.

In 10 patients (3.4% of total; 14.9% of abnormal), we identified a (likely) pathogenic variant (SNV and/or CNV) as monogenic cause for their infertility (Table 1B and Figure 3). Four patients with an extreme (oligo)asthenozoospermia had one homozygous or two heterozygous pathogenic variant(s) in MMAF‐associated genes (*CFAP43*, *CFAP69*, *DNAI1*, and *FSIP2*). In two patients, a causal variant was found that could explain their azoospermia (heterozygous variant in *NR5A1* and hemizygous variant in *RBBP7*). In one patient with macrozoospermia, a homozygous pathogenic variant was identified in *AURKC*. In one other patient with azoospermia, two compound heterozygous deletions in the long arm of chromosome 15 (15q15.3) encompassing *CATSPER2* were detected (the recurrent deletion of both *STRC* and *CATSPER2* ^25^ and an atypical smaller deletion with only *CATSPER2* involved), effectively resulting in a homozygous deletion of *CATSPER2* that is associated with asthenoteratozoospermia and not azoospermia. This individual is a heavy smoker (one package of cigarettes per 1–2 days), which could have influenced the semen motility, but not primarily semen production. In two patients, with globozoospermia, pathogenic variants in the *DPY19L2* gene were found: one with a homozygous deletion of the complete gene and one with a combination of a heterozygous deletion of the *DPY19L2* gene and a hemizygous frameshift variant. The respective couples were offered to proceed with ICSI treatment in combination with artificial oocyte activation.

Furthermore, as shown in Table SIII and Figure 3, we identified one or more single nucleotide or copy number variant(s) of unknown significance (VUS) in genes associated with male infertility in 12 patients (4.1% of total; 17.9% of abnormal). Further investigation by, for example, segregation analysis for phasing and/or RNA analysis is required to better understand the role of all these variants in the infertility phenotype.

In one patient (P123), a Robertsonian translocation (45,XY,der(13;14)(q10;q10)) was identified by karyotyping; ES was normal in this person.

## 4 DISCUSSION

We present a validation study to first‐tier application of ES with simultaneous CNV and SNV analysis to replace current (standard) diagnostic genetic testing in male infertility (“mandatory” aberrations previously detected with karyotyping, AZF deletion screening, and *CFTR* analysis in case of CBAVD suspicion). Furthermore, the results of a 10‐month evaluation of this new strategy in 292 infertile men are presented. We determined that ES with simultaneous CNV and SNV analysis could detect the “mandatory” aberrations with a sensitivity and specificity of >95%. In the first 10 months after implementation of this approach in our clinical diagnostics routine, we identified one or more (potentially) clinically relevant genetic anomalies in 22.9% (67 of 292) of all cases, among which 30 of 67 patients (10.2% of total; 44.8% of abnormal) with aberrations that could have been detected with the current standard methods (chromosomal anomalies including 47,XXY, AZF deletions, and *CFTR* variants present in the Elucigene CF‐EU2v1 kit). In 28 of 67 patients (9.6% of the total; 41.8% of abnormal) a (possible) (mono)genetic cause was identified with ES that could not be identified with standard methods (extra diagnostic yield). In nine patients (3.1% of the total; 13.4% of abnormal), carriership of CF was found, for which carrier screening in the partner was recommended before the MAR trajectory was continued.

### 4.1 Exome sequencing for the detection of AZF deletions

Currently, most laboratories use STS‐PCR to detect AZF deletions as recommended in the European Academy of Andrology (EAA)/ European Molecular Genetics Quality Network (EMQN) best practice guidelines for molecular diagnosis of Y‐chromosomal microdeletions.^24^, ^26^ The STS sites are used as proxies for regions that are thought to be crucial for spermatogenesis, but the spermatogenic defect is likely to be caused by a deletion of genes located in the critical regions.^27^ The current guidelines recommend testing of at least two STS sites per AZF region, but we demonstrate that analysis of the presence of two genes per AZF region is also sufficient for the reliable detection of complete AZFa, AZFb, and AZFc deletions. In some cases, ES can even improve the detection of AZF deletions: an example of this is VAL14. This individual was previously diagnosed with a classic interstitial AZFc (b2/b4) deletion (sY160(+)), but exome CNV analysis showed an additional loss of both *PRY* copies, which are typically unaffected by AZFc deletions,^28^ indicating an alternative proximal breakpoint. The clinical significance of this alternative breakpoint and the additional loss of the *PRY* copies remains as yet unclear.

An additional benefit of using ES instead of STS‐PCR is the detection of pathogenic SNVs and CNVs in genes located in the AZF regions. Currently, due to technological limitations, reliable SNV and CNV detection is only possible in the single‐copy AZF region genes (including *UPS9Y*, *DDX3Y*, *KDM5D*, *EIF1AY*, and *RPS4Y2*). Most of the genes in the AZF regions are strong candidate genes for involvement in male infertility,^29^ but it remains largely unclear which individual gene or combination of gene deletions in the AZF regions actually cause spermatogenic failure. The only region this has been clarified for is the AZFa region, for which the key gene loss leading to spermatogenic failure is *DDX3Y*. ES studies have already reported pathogenic SNVs in this gene showing an additional benefit of screening for SNVs in AZF genes.^30^

### 4.2 ES for detection of (sex) chromosomal anomalies

Our results show that ES is a reliable method to detect non‐mosaic (sex) chromosomal anomalies, including Klinefelter syndrome and XX‐males. A particular finding in our validation study was in sample VAL6, which was previously diagnosed with a complete AZFbc (P5/distal P1) deletion (sY160(+)) and chromosomal analysis showing a 46,XY karyotype. The ES CNV profile, however, not only showed the complete AZFbc deletion, but was also highly indicative of a 46,X,idic(Y)(q11.222) karyotype, which was an improvement compared with standard methods. Array analysis (CytoScan HD, Thermo Fisher Scientific) confirmed the ES CNV profile. Upon further inspection of the original STS‐PCR results and analyzed metaphases, a 46,X,idic(Y)(q11.222) pattern indeed seemed to be fitting. No FISH analysis could be executed to confirm the isodicentric nature of the Y‐chromosome.

For the determination of the breakpoint(s) of Y‐chromosomal anomalies, the use of ES has both advantages and disadvantages. In sample VAL36, the breakpoint could be determined more accurately (46,X,idic(Y)(q11.2) changed to 46,X,idic(Y)(q11.221)). In samples VAL37, VAL38, and VAL39, ES was less accurate in detecting the approximate breakpoint location. Due to the lack of exome enrichment distal to the centromere (no enrichment for chrY:9368285‐14517915 (GRCh37/hg19)), it remains impossible to determine the difference between an iso(Yp) or idic(Yq) chromosome with a breakpoint upstream of the AZFa region. Even though these are genetically different, there is no clinically relevant difference between an iso(Yp) or idic(Yq), as both cause Sertoli‐cell only syndrome and meiotic arrest, and in both cases testicular sperm extraction (TESE) treatment is not recommended. Routine cytogenetic analysis (karyotyping, and if needed, FISH) can be used to elucidate the correct karyotype.

Interestingly, we identified one case of 45,X/46,XY (VAL84) in the subset of negative male controls. According to national recommendations at least five metaphases should be karyotyped in male infertility.^31^ Extension to screening of 30 cells is only recommended when ≥1 abnormal cell is detected. This means that low‐grade mosaicisms (<20%) may remain undetected. In this specific patient (with oligozoospermia), the analyzed metaphases all showed a normal 46,XY karyotype, but with ES a loss of the Y chromosome in ∼30% of the DNA was identified. No metaphases were available anymore to extend the chromosomal analysis and confirm this mosaicism in cultured peripheral blood lymphocytes. The results were shared with the patient by his physician, and cardiological and endocrinological follow‐up showed no abnormalities.

This result indicates that ES for male infertility is likely to be more sensitive in detecting mosaic sex chromosomal aneuploidies than the current karyotyping protocol. Although, it remains impossible to accurately determine the grade of mosaicism, with the exome analysis methodologies currently used in our laboratory, based on experience, the detection limit is probably between 10% and 20% for numerical chromosomal anomalies.

### 4.3 Exome sequencing for the detection of clinically relevant variants in the *CFTR* gene

Previously, in routine diagnostics in our laboratory, patients with (suspected) CBAVD were tested with the Elucigene CF‐EU2v1 kit, which contains the 53 most common Caucasian *CFTR* variants, including the T/TG tract in intron 9.^22^ Extended analysis of the complete *CFTR* gene using Sanger sequencing and/or MLPA was only performed in patients with non‐Caucasian heritage or if requested by the physician in case only one variant had been identified with the Elucigene kit. We and others already showed that in CBAVD patients frequently a rare second variant, not present in the Elucigene CF‐EU2v1 kit, can be identified *in trans* with a common Caucasian one.^21^ At least one of those rare variants was detected in the *CFTR* gene in three of 10 patients in our evaluation cohort (Table 2). In two of these patients, none of the variants were present in the kit and without ES, the cause of the azoospermia would not have been identified, but also the corresponding carriership for CF would not have been recognized.

**TABLE 2:** Results of physical examination, semen analysis, and genetic analysis in patients diagnosed with *CFTR*‐related CBAVD.

Patient	Physical examination vas deferens	Semen analysis			CFTR variants
		Result	Volume	pH	
**Both *CFTR* variants included in the Elucigene CF‐EU2V1 kit**					
P62	Not palpable	Azoospermia	0.1 mL	Unknown	*CFTR* Chr7(GRCh37):g.117171029G>A NM_000492.4:c.350G>A p.(Arg117His); hom
P70	Not palpable	Azoospermia	0.4 mL	Unknown	*CFTR* Chr7(GRCh37):g.117171029G>A NM_000492.3:c.350G>A p.(Arg117His); het *CFTR* Chr7(GRCh37):g.117267591C>T NM_000492.4:c.3484C>T p.(Arg1162^a^); het
P104	Both palpable	Azoospermia	1.5 mL	6.4	*CFTR* Chr7(GRCh37):g.117199646_117199648del NM_000492.4:c.1521_1523del p.(Phe508del); het *CFTR* Chr7(GRCh37):g.117171029G>A NM_000492.3:c.350G>A p.(Arg117His); het
P113	Both doubtful palpable	Azoospermia	0.7 mL	6.4	*CFTR* Chr7(GRCh37):g.117199646_117199648del NM_000492.4:c.1521_1523del p.(Phe508del); het *CFTR* Chr7(GRCh37):g.117188684T>G NM_000492.4:c.1210‐11T>G p.?; het (5T/12TG)
P115	Not palpable	Azoospermia	1.0 mL	Unknown	*CFTR* Chr7(GRCh37):g.117199646_117199648del NM_000492.4:c.1521_1523del p.(Phe508del); het CFTR Chr7(GRCh37):g.117171029G>A NM_000492.3:c.350G>A p.(Arg117His); het
P139	Left: doubtful palpable Right: present, but thin	Azoospermia	0.5 mL	6.0	*CFTR* Chr7(GRCh37):g.117199646_117199648del NM_000492.4:c.1521_1523del p.(Phe508del); het *CFTR* Chr7(GRCh37):g.117188684T>G NM_000492.4:c.1210‐11T>G p.?; heterozygous (5T/12TG)
P257	Left: doubtful palpable Right: present	Azoospermia	0.35 mL	6.4	*CFTR* Chr7(GRCh37):g.117171029G>A NM_000492.4:c.350G>A p.(Arg117His); het *CFTR* Chr7(GRCh37):g.117199646_117199648del NM_000492.4:c.1521_1523del p.(Phe508del); het
**At least one *CFTR* variant NOT included in the Elucigene CF‐EU2V1 kit**					
P197	Suspicion of blind ending vas deferens, both sides only 2 cm palpable	Azoospermia	1.1 mL	6.0	*CFTR* Chr7(GRCh37):g.117175339T>G NM_000492.3:c.617T>G p.(Leu206Trp); het^a^ *CFTR* Chr7(GRCh37):g.117232693del NM_000492.4:c.2472del p.(Asn825fs); het^a^, ^b^
P207	Unknown	Azoospermia	Unknown		*CFTR* Chr7(GRCh37):g.117171090_117171092dup NM_000492.4:c.413_415dup p.(Leu138dup); hom^a^, ^b^
P268	doubtful palpable	Azoospermia	0.3 mL	6.4	*CFTR* Chr7(GRCh37):g.117188852T>C NM_000492.4:c.1367T>C p.(Val456Ala); hom^a^, ^b^

^a^ Variant associated with mild form of cystic fibrosis.

^b^ Variant not present in Elucigene CF‐EU2V1 kit; het, heterozygous; hom, homozygous.

More importantly, compound heterozygous variants in the *CFTR* gene can be present in patients without a diagnosis of CBAVD based on physical examination,^21^ which requires much expertise and even then, is not always certain. In our evaluation study, in only three of nine patients of whom clinical information was available, a clear absence of both vas deferens was detected upon physical examination. However, in all, semen analysis showed low pH (<7.2) and/or low volume (<1.5 mL) next to the azoospermia, fitting an obstruction in of the absence of the vas deferens (Table 2). The combined results of physical examination (even with doubtful palpable vas deferens), semen analysis (azoospermia, low pH, and low volume), and genetics (two variants in *CFTR*) can firmly establish the diagnosis of CFTR‐related CBAVD. Clinical re‐evaluation for other symptoms of CFTR‐related disorders (i.e., bronchiectasis or pancreas insufficiency) or mild CF is indicated in these patients.^22^

### 4.4 Implementing exome sequencing as a first‐tier test in male infertility genetic diagnostics

Of the 67 patients with clinically relevant variants identified with ES, in 30 this variant would have been detected with routine analysis as described in the current guidelines as well (unbalanced (sex) chromosomal anomalies including Klinefelter, AZF deletions, *CFTR* variants in the Elucigene CF‐EU2v1 kit; Tables 1A and 2). In a retrospective study of the diagnostic results of 1222 infertile men investigated in our laboratory between 2014 and 2018, we found a clinically relevant variant in 10.6% (data not shown), comparable to the 10.2% we identified in our evaluation cohort. In that same retrospective study, ∼1% of the investigated males had a balanced translocation (13 of 1222). Routine karyotyping was executed in parallel in 252 of 292 patients of the evaluation cohort and in two cases (two of 252; 0.8%), a balanced translocation was detected. Based on karyotype analysis, P241 appeared to have a balanced complex translocation (45,XY,der(4)t(4;21)(p10;q10),der(22)t(4;22)(q10;q10)). Using CNV analysis of the ES data, however, we determined that the translocation was in fact unbalanced: multiple losses around the centromere of chromosome 4 were recognized, but the clinical significance of these losses remain unclear. This is in line with previous studies which showed that 25%–50% of microscopically balanced reciprocal translocations reveal cryptic genomic imbalances.^32^, ^33^ The other patient with a microscopically balanced abnormality (P123) carried a Robertsonian translocation (45,XY,der(13;14)). About 40% of the balanced translocation carriers identified in the context of male infertility in our laboratory have a Robertsonian translocation (personal data not shown). As expected, the der(13;14) seen in P123 did not result in an abnormal ES CNV profile: balanced Robertsonian translocations so far cannot be recognized with any (short‐ or long‐read) next‐generation sequencing technique or with optical genome mapping, due to highly repetitive centromeric regions of the acrocentric chromosomes. As the presence of a balanced (Robertsonian) translocation may result in increased risk for the couple for having miscarriages or affected live‐born children, and special treatment options within the MAR should be recommended (i.e., pre‐implantation genetic testing); consequently, for the time being, karyotyping remains necessary. The detection rate of CNVs and structural variation including reciprocal translocations can be further improved by performing whole genome sequencing, due to improved structural variant calling and breakpoint determination at bp resolution.^34^, ^35^

SNV and CNV analysis of genes associated with male infertility increased the diagnostic yield compared with standard methods with 6.8%, as in our evaluation study a monogenetic cause was found in 20 of 292 men with CBAVD, azoospermia, oligo‐, astheno‐, and/or teratozoospermia (including MMAF, macrozoospermia, and globozoospermia).

In terms of costs, a combination of ES and karyotype analysis in our laboratory setting is similar to a combination of AZF deletion screening, *CFTR* common variant testing and karyotype analysis (the estimation is that the difference in price will be lower than 10%). As the diagnostic yield was almost doubled (10.2%–19.8%, excluding CF carriers), the cost‐effectiveness of our ES strategy is higher. Furthermore, we demonstrate that ES can be used to detect sex chromosomal anomalies, including 47,XXY. To further optimize the cost‐effectiveness, performing karyotype analysis sequentially, rather than in parallel with ES, would make the need for karyotype analysis redundant in at least 14% of the cases (cause identified *n* = 43 of 292, of which 17 with 47,XXY or another sex chromosomal anomaly), while delaying the diagnosis of a balanced translocation in 0.68% (*n* = 2 of 292). Delaying karyotype analysis would require a quick turnaround time of both ES and karyotype analysis, as often the fertility treatment procedure has already been initiated in the female partner. The turnaround time of genetic tests can differ significantly between laboratories. In the Netherlands, genetic testing for infertility is preferably completed in 4–6 weeks. Sequential testing would increase the turnaround time to at least 8–12 weeks. To further reduce the number of tests per patient, karyotyping can be omitted for patients with a sperm phenotype which is not related to chromosomal abnormalities such as globozoospermia.

The accessibility to ES varies greatly between countries. Decreasing sequencing costs^36^ will improve access for diagnostic laboratories to NGS‐based methods, including access through commercial sequencing providers (sometimes even offering interpretation and reporting as well). Consequently, ES‐based approaches for male infertility will increasingly become part of standard care.

Although we were able to identify a possible or definitive genetic cause of infertility in over 20% of the patients, still in 79.8% (233 of 292) of them, including the CF carriers, no genetic cause for the male infertility was found with ES or karyotyping. As the number of genes associated with male infertility increases each month,^7^ re‐analysis of the ES data in the (near) future is likely to elucidate a monogenetic cause in at least a number of these cases. Due to the extreme heterogeneity of genetic causes of male infertility, the list of genes associated with male infertility will become manifold larger.^37^ Early adaptation to exome or genome sequencing, rather than more targeted approaches, allows for rapid modification of the investigated gene panels. This study can be used as guidance for validating and implementing an NGS‐based test for male infertility.

### 4.5 Opportunities for identifying co‐morbidities associated with monogenic causes of severe male infertility

Identifying causal genetic variants provides opportunities for clinical re‐evaluation and identification of co‐morbidities besides the infertility. Examples of such additional implications are shown in P179, who had many upper airway infections and nasal polyps as a child and is now diagnosed with PCD due to two heterozygous pathogenic variants in the *DNAI1* gene. Likewise, in case P197, with a suspicion of blind ending vas deferens, in whom two heterozygous likely pathogenic variants in the *CFTR* gene were identified, in retrospect had a family history of CF (affected siblings of one of his parents) and mild CF symptoms himself.

In 15 other patients (5.1%), a VUS was disclosed in one of the genes of the male infertility gene panel or beyond, for which segregation analysis and/or RNA analysis is indicated to further investigate the pathogenicity.

The added value of generating sequencing data of the complete exome was shown in three patients with an additional clinical phenotype for whom an extra bioinformatic gene panel filter could be applied, and potentially clinically relevant variants were identified. In one patient (P153) with a suspicion of PCD, a combination of a pathogenic variant and a VUS in the *TTC12* gene, associated with PCD type 45 (Online Mendelian Inheritance in Man [OMIM] phenotype #618801), were identified. As it was recently shown that *TTC12* variants can also cause asthenoteratozoospermia without a PCD phenotype,^38^ this gene was added to our most recent male infertility gene panel version. Another case in whom analysis beyond the male infertility gene panel was helpful, was an infertile man (P25) with a sister with primary ovarian insufficiency (POI) and polyposis. In both this male and his sister, a compound heterozygous pathogenic variant and VUS were revealed in the *HROB* gene, which encodes a protein that recruits the MCM8–MCM9 helicase to sites of DNA damage to promote DNA synthesis. Variants in the genes in this helicase have been clearly associated with POI^39^ and are thought to predispose to mixed polyposis.^40^, ^41^ In the third patient (P89), with azoospermia and hypogonadotropic hypogonadism, a VUS in the *CCDC141* gene was seen.

## 5 CONCLUSION

ES with combined SNV and CNV analysis is a reliable first‐tier method to detect the most common genetic causes in male infertility. The diagnostic yield in our cohort was higher compared with the current strategy recommended in guidelines, as our ES‐based strategy identifies genetic causes not detectable by current (standard) methods and is available for semen abnormalities other than (non‐)obstructive azoospermia and extreme oligozoospermia. A genetic diagnosis helps professionals in counseling for treatment, possible co‐morbidities as well as risk for offspring or family members.

As the detection limit for low‐grade mosaicisms is not clear yet and balanced translocations, especially Robertsonian translocations, cannot be detected with ES, parallel or sequential karyotyping will remain necessary to conform to current guidelines.

## AUTHOR CONTRIBUTIONS

Manon S. Oud and Dineke Westra performed the validation and evaluation study, analyzed the results, and wrote the first draft. Nicole de Leeuw and Brigitte H. W. Faas designed the validation and evaluation study. Dominique F. C. M. Smeets designed the setup of the validation study. Liliana Ramos and Godfried W. van der Heijden performed the semen analysis. Raoul G. J. Timmermans and Maartje van de Vorst developed bioinformatic tools for CNV detection. Tom Hofste supervised the sequencing. Marlies J. E. Kempers, Marijn F. Stokman, and Kathleen W. M. D'Hauwers conducted patient counseling. All the authors discussed the results, critically revised the manuscript, and approved the final version.

## CONFLICT OF INTEREST STATEMENT

The authors declare no conflicts of interest.

## Supporting information

Supporting Information

Supporting Information

## Data Availability

The data that support the findings of this study are available on request from the corresponding author. The data are not publicly available due to privacy or ethical restrictions.
