# Supplementary material for: Innovative all‐in‐one exome sequencing strategy for diagnostic genetic testing in male infertility: Validation and 10‐month experience
Source: Andrology. 2024 Aug 24;13(5):1078–92. doi: 10.1111/andr.13742 (PMC12183012; doi:10.1111/andr.13742)
Supplement: Supplementary file 1 — Supporting Information [file ANDR-13-1078-s001.docx]

Diagnostic analysis of the exome data for the indication male infertility was performed in 5 steps:

1. The YX coverage ratio, routinely used by our laboratory as a quality control check by comparing the reported sex on the test request form to the determined genetic sex (genetic male: YX ratio > 1.0; genetic female: YX ratio <0.2), provides an indication about abnormal ratios between X and Y chromosomal material. A ratio between 0.2 and 1.0 indicates a sex chromosomal anomaly such as 46,XX(SRY+) male, 47,XXY or an AZFbc deletion. Ratio’s well above 1.5 indicate an excess of Y chromosomal material, as is the case in 47,XYY.
2. A representation of the karyotype is generated by an in-house tool called Karyovisualizer (unpublished). This virtual karyotype is inferred from CoNIFER and ExomeDepth CNV calls, as well as large stretches of homozygous regions based on SNV calls. This allows for the detection of gross chromosomal anomalies such as aneuploidies, unbalanced translocations affecting genes on any chromosome, uniparental disomies (isodisomy) and/or consanguinity.
3. The CoNIFER calls on chromosome X and the ExomeDepth calls on chromosome X and Y are further inspected in the Integrative Genomics Viewer (IGV) to detect mosaic gross anomalies (partially) undetected by ExomeDepth such as 46,XY/45,X, and microdeletions on chromosome Y. This analysis included visual inspection of at least 6 genes in the AZFa, -b and -c regions on chromosome Y to detect classical AZF deletions (AZFa: *USP9Y* and *DDX3Y*; AZFb: *EIF1AY* and *RPS4Y2*; AZFc: *BPY2* and *DAZ4*) (see figure 2 and Supplemental Table SV). If an AZF deletion is detected, an extension analysis is performed to elucidate the size of the deletion (*XKRY* for AZFa, *HSFY1* for AZFb (distinguishing between a P4 or P5 breakpoint), *PRY2* (distinguishing between a classical or atypical AZFc deletion) and heterochromatin position chrY:58911807-58912041 (distinguishing between interstitial or terminal deletion) for AZFc).
4. The CoNIFER and ExomeDepth CNV calls affecting genes in the male infertility gene panel or affecting >2 kb of chromosome Y were inspected and classified according to the ACMG guidelines.
5. SNV calls within our gene panel were inspected and classified while taking into account the findings in the CNV analysis for potential compound heterozygosity of an SNV and a CNV in the same gene.
